# Variation of Major Chemical Composition in Seed-Propagated Population of Wild Cocoa Tea Plant *Camellia ptilophylla* Chang

**DOI:** 10.3390/foods12010123

**Published:** 2022-12-26

**Authors:** Xin-Qiang Zheng, Shu-Ling Dong, Ze-Yu Li, Jian-Liang Lu, Jian-Hui Ye, Shi-Ke Tao, Yan-Ping Hu, Yue-Rong Liang

**Affiliations:** 1 Tea Research Institute, Zhejiang University, Hangzhou 310058, China; 2 Tea Research Institute of Pu’er City, Pu’er 665000, China

**Keywords:** tea plant, seedling, catechins, caffeine, amino acids

## Abstract

Excessive intake of high-caffeine tea will induce health-related risk. Therefore, breeding and cultivating tea cultivars with less caffeine is a feasible way to control daily caffeine intake. Cocoa tea (*Camellia ptilophylla* Chang) is a wild tea plant which grows leaves with little or no caffeine. However, the vegetative propagation of cocoa tea plants is difficult due to challenges with rooting. Whether natural seeds collected from wild cocoa tea plants can be used to produce less-caffeinated tea remains unknown, because research on the separation of traits among the seed progeny population is lacking. The present study was set to investigate the variation of caffeine and other chemical compositions in seed-propagated plant individuals using colorimetric and HPLC methods. It shows that there were great differences in chemical composition among the seed-propagated population of wild cocoa tea plants, among which some individuals possessed caffeine contents as high as those of normal cultivated tea cultivars (*C. sinensis*), suggesting that the naturally seed-propagated cocoa tea seedlings are not suitable for directly cultivating leaf materials to produce low-caffeine tea. Therefore, the cocoa tea plants used for harvesting seeds for growing low-caffeine tea plants should be isolated in order to prevent their hybridization with normal cultivated *C. sinensis* plants. Interestingly, the leaves of cocoa tea seedlings contained high levels of gallocatechin gallate (GCG) and would be a good source of leaf materials for extracting more stable antioxidant, because GCG is a more stable antioxidant than epigallocatechin gallate (EGCG), the dominant component of catechins in normal cultivated tea cultivars. Some plant individuals which contained low levels of caffeine along with high levels of amino acids and medium levels of catechins, are considered to be promising for further screening of less-caffeinated green tea cultivars.

## 1. Introduction

Tea has attracted great attention owing to its abundant polyphenolic antioxidants which contribute to the promotion of health benefits and the prevention of many chronic diseases [1,2]. However, tea leaves from the majority of tea cultivars contain high levels of purine alkaloids, among which caffeine is the most abundant [3,4]. Moderate intake of caffeine from tea shows physiological effects such as alleviating mental fatigue and increasing energy and alertness [5]. However, high consumption of tea containing high levels of caffeine will induce a number of health-related risks [6], such as increased blood pressure, gastrointestinal disturbances, palpitations, anxiety, tremors, and insomnia [7,8,9]. Excessive intake of high-caffeine tea by pregnant women can lead to birth defects in babies and produce infertility [5,10]. Tea is one of the most prominent sources of daily caffeine intake [4,11,12], and drinking less-caffeinated teas will reduce this daily caffeine intake [3,13,14].

The caffeine contents in traditional green tea and black tea ranged from 1.43% to 3.48% by dry weight (DW) [12]. Low-caffeine tea can be obtained by removing caffeine from traditional tea leaves by selective extraction [15,16,17], developing caffeine-free wild tea plants [18,19], and improving tea cultivars using biotechniques such as overexpressing caffeine-degradative pathway genes or silencing caffeine biosynthesis pathway genes [6]. Though less-caffeinated tea products can be obtained by decaffeination processing techniques, the resulting teas are considered to be artificial, and bioactive compounds such as catechins and theanine were partially removed [15,17]. There is a long way to go to breed less-caffeinated tea cultivars using biotechnology, because it is now technically difficult to regenerate tea plants from tea cells [20]. The caffeine concentrations in cultivated tea cultivars were found to be significantly higher than those in wild tea plants [18]. There was a great difference in the caffeine content between wild tea plants, ranging from 0 to 8.39% (DW) [21,22]. It is feasible to produce natural less-caffeinated tea using screened low-caffeine wild tea plants such as cocoa tea (*Camellia ptilophylla* Chang) [23,24]. However, wild tea plants are limited in quantity and cannot meet the needs of mass production of less-caffeinated tea products [25,26]. It is necessary to increase the yield of these wild tea plants by artificial cultivation in order to meet production demands. However, it is difficult to propagate these wild tea plants by vegetative cutting like the normal tea cultivars, because the cuttings do not root properly [27]. The contents of tea polyphenols in clonal cocoa tea cultivars No. 1 and No. 2 were 32.21% and 29.17%, with 2.26% and 2.27% amino acids, and 24.15% and 22.56% total catechins, respectively, but no caffeine [28]. The cocoa tea cultivars No. 1 and No. 2 were screened from wild *C. ptilophylla* plants, and they were difficult to propagate by cutting [29]. The separation of caffeine content in the population of seed-propagated seedlings had great impact on the caffeine levels of the harvested leaves, however, this issue remains unresolved. The present study was set to investigate the separation of major tea chemical components, including caffeine, in the seed-propagated population of wild, less-caffeinated cocoa tea plants, which would provide useful information for propagating less-caffeinated *C. ptilophylla* plants. 

## 2. Materials and Methods

### 2.1. Materials

The tea seeds used in the study were collected from less-caffeinated wild tea plants (*Camellia ptilophylla*) in the Nankun Mountains in Longmen County, Guangdong Province, China in October 2019. The tea seeds were sown in a nursery field in February 2020, and the seedlings were transplanted to tea fields at a row spacing of 1.5 m and a plant spacing of 0.5 m in November 2020. The field management and fertilization of these tea plants were the same as production tea fields. Tea shoots with two leaves and a bud were plucked on 39 plants obtained in early May, 2022, then fixed in a microwave oven (700 W, Midea Group Co., Ltd., Foshan, China) for 1 min, and dried at 80 °C to <5% (*w/w*) moisture content. The dried leaves were ground and sifted through a 12-mesh sifter and stored at 4 °C until use.

HPLC reference compounds including (−)-epigallocatechin gallate (EGCG), (−)-epigallocatechin (EGC), (−)-epicatechin gallate (ECG), (−)-epicatechin (EC), (+)-catechin (C), (+)-gallocatechin gallate (GCG), (+)-gallocatechin (GC), (+)-catechin gallate (CG), caffeine, theacrine, theobromine, and theophylline were purchased from Sigma-Aldrich (St. Louis, MO, USA). The Folin–Ciocalteu reagent, the gallic acid for determining the total polyphenols, and the ninhydrin for determining free amino acids were also Sigma-Aldrich products.

### 2.2. Methods

#### 2.2.1. Extraction of Tea leaves

The extraction of tea leaves was carried out according to the methods of a previously published paper [15]. That is, the ground tea sample (0.15 g) was extracted in a glass tube containing 25 mL 50% (*v*/*v*) ethanol solution for 20 min, during which time the tube was vortexed for 1 min at the 10th min and the 20th min. The extract was centrifuged for 15 min at 12,000 rpm and 4 °C, and the supernatant was collected for chemical analyses.

#### 2.2.2. Determination of Total Polyphenols Content

**Figure 1:**
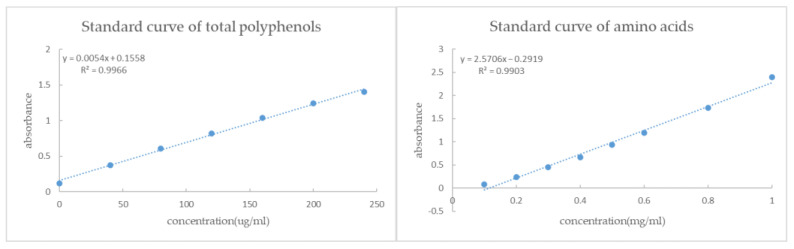
Standard curves of total polyphenols (**left**) and amino acids (**right**).

The total polyphenols content was determined by Folin–Ciocalteu method using EGCG as a calibrated reference substance based on the published paper [30]. A total of 1.0 mL of tea extraction solution and 5.0 mL of 10% (*v*/*v*) Folin–Ciocalteu reagent were transferred to a 10 mL volumetric flask, shaken well, and then 4 mL of Na_2_CO_3_ solution (75.0 mg/mL) was added, mixed completely by shaking, and the product stood for 60 min at room temperature. The absorbance of the reacted solution was determined at 765 nm on a HP8453E UV-VIS spectrophotometer (Hewlett Packard Company, Palo Alto City, CA, USA). In the present study, EGCG was used as the calibrated reference compound, and the total polyphenols content was expressed in terms of EGCG equivalent (Figure 1).

#### 2.2.3. Determination of Total Amino Acids Content

The concentration of amino acids was determined by the ninhydrin assay method [31]. A total of 2 mL of the above tea extraction solution was transferred to a 50 mL volumetric flask with 1 mL of reagent (20 g/L of ninhydrin and 0.8 g/L of SnCl_2_·2H_2_O) and 1 mL of buffer (0.067 M Na_2_HPO_4_ and 0.067 M KH_2_PO_4_, pH 8.0) and reacted for 15 min in a boiling water bath. The control flask contained 2 mL of distilled water, 1 mL of reagent, and 1 mL of buffer. The reacted solution was then transferred to a quartz cell with a black aperture (1 cm light-path) and colorimetric measurement was made on an HP8453E UV-VIS spectrophotometer (Hewlett Packard Company, Palo Alto City, CA, USA) at a wavelength of 570 nm. Theanine (Sigma-Aldrich, St. Louis, MO, USA) was used as the amino acid reference to make the calibration graph, and the amino acid concentration of the tea samples was expressed in terms of theanine equivalent based on its absorbance at 570 nm on the calibration graph (Figure 1).

#### 2.2.4. Determination of Alkaloids and Catechins

**Figure 2:**
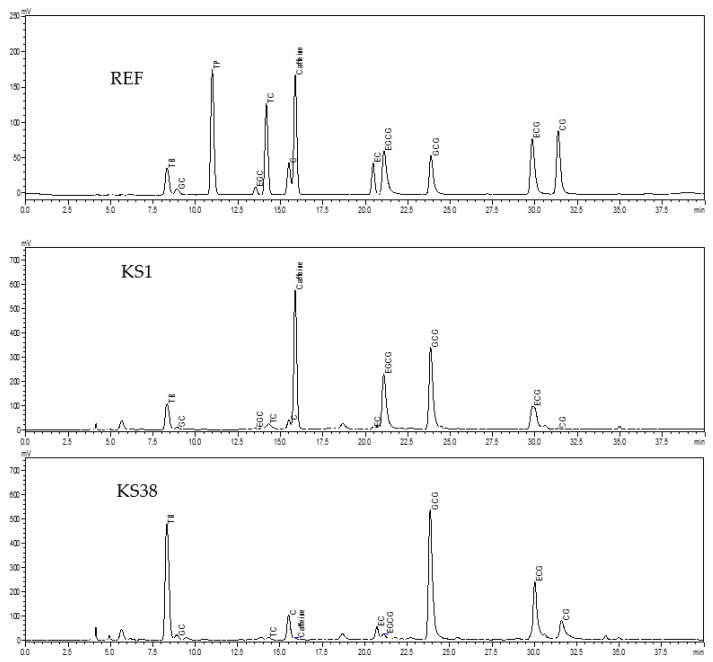
The HPLC chromatograms of references compounds (REF), samples KS1 and KS38.

The contents of individual alkaloids and catechins were determined following the HPLC method and using an HPLC-20AD System (Shimadzu, Kyoto, Japan) under the following conditions and according to our previous paper [32]. injection volume: 10 µL; column: TC-C18 5 μm, 4.6 × 250 mm (Agilent Technologies Inc., Santa Clara, CA, USA); oven temperature: 35 °C; gradient elution: linear gradient increasing from 18% mobile phase B to 80% mobile phase B in 35 min; flow rate: 1 mL/min; mobile phase A: acetonitrile/acetic acid/water (6/1/193, *v*/*v*/*v*), mobile phase B: acetonitrile/acetic acid/water (60/1/139, *v*/*v*/*v*); detection wavelength: 280 nm. The concentrations of the individual alkaloids and catechins were identified by comparing the chromatographic retention time and peak area with authentic reference compounds (Figure 2). The alkaloids and catechins contents in the dry tea leaves were calculated as follows; the results were on the basis of the dry weight of the leaf.

$$W_{1}=\frac{S_{1}\times C_{1}\times V}{Si_{1}\times m}$$

$$W_{2}=\frac{S_{2}\times C_{2}\times V}{Si_{2}\times m}$$

Formula: *W*_1_, content of alkaloids in sample (mg/g); *W*_2_, content of catechins in sample (mg/g); *S*_1_, peak area of alkaloids in sample extraction solution; *S*_2_, peak area of catechins in sample extraction solution; *Si*_1_, peak area of alkaloids in standard measuring solution; *Si*_2_, peak area of catechins in standard measuring solution; *m*, sample weighing mass (g); *C*_1_, concentration of alkaloids in standard measuring solution (mg/mL); *C*_2_, concentration of catechins in standard measuring solution (mg/mL); *V*, solvent volume used for tea extraction (mL).

#### 2.2.5. Statistical Analyses

Quantificational analysis was conducted based on the retention time and peak areas of the authentic reference compounds. All of the tests were repeated three times, and the results were expressed as the mean ± standard deviation. Statistical analysis was performed using SPSS version 26.0 (SPSS, Chicago, IL, USA). 

## 3. Results

### 3.1. Contents of Total Polyphenols and Amino Acids

The total polyphenols content in the 39 plants ranged from 18.32 ± 0.34% (DW) to 36.03 ± 0.95% (DW), with a mean of 29.42 ± 3.88% (DW) and a coefficient of variation (CV) of 13.20%. In green tea, the polyphenol content ranged from 12% to 23% [31]. This study showed that the polyphenol contents in the cocoa tea plants, except for KS36 and KS58, were more than 23%, suggesting the cocoa tea plants are abundant in polyphenolic compounds. The total amino acids content in these cocoa tea plants ranged from 3.25 ± 0.03% to 10.40 ± 0.33%, with a mean of 6.24 ±1.63% and a CV of 26.10% (Table 1). Based on the CV, the variation of amino acids was much broader than that of the tea polyphenols. The amino acids contents in green tea were 3–6% [31]. Table 1 shows that 23 plants contained more than 6% amino acids, and no plants had less than 3%, suggesting that cocoa tea plants contained high levels of amino acids. It is particularly interesting that some plants—KS17, KS23A, KS41, KS55B, and KS65—contained more than 8% amino acids, which was much higher than normal tea cultivars [31,32].

### 3.2. Contents of Alkaloids

Four alkaloids including caffeine, theacrine, theobromine, and theophylline, were detected, and the total alkaloids content in the 39 plants ranged from 27.03 ± 0.90 mg/g to 70.45 ± 4.69 mg/g, with a mean of 48.79 ±10.70 mg/g (DW) and a CV of 21.94%. Among the alkaloids, theobromine, with a mean 25.26 mg/g, was the most abundant based on total mean value, followed by caffeine with a total mean volume of 20.07 mg/g (Table 2). Theophylline was the least abundant, and it was detected in seven plant individuals (18% of total plants). For caffeine, the variation was great. The highest content was higher than 50 mg/g; 12 plants contained 35–50 mg/g of caffeine, 8 plants contained 20–35 mg/g of caffeine, 5 plants contained no caffeine, and 13 plants had a caffeine level less than 5 mg/g, the maximum limit for commercial decaffeinated tea products. These results suggest that the caffeine content of seed-propagated plants of wild cocoa tea separated greatly, among which about 50% of the plant individuals met the requirements of low-caffeine tea production. 

### 3.3. Contents of Catechins

The content of total catechins in the detected samples ranged from 70.58 mg/g (KS8) to 316.79 mg/g (KS38), with the mean being 186.85 mg/g and the CV being 25.75% (Table 3). Based on the mean values, gallocatechin gallate (GCG) was the most abundant among the detected catechins, unlike normal tea cultivars in which epigallocatechin gallate (EGCG) is the most abundant component of catechins (32). Epicatechin (EC) and epigallocatechin (EGC) were the two least-abundant components of catechins, with epicatechin gallate (ECG), catechin (C), gallocatechin (GC), and catechin gallate (CG) ranging in between (Table 3). 

## 4. Discussion

It is known that caffeine has a chemical similarity with adenine which blocks the adenosine receptors in nerve cells, and so it acts as a central nervous system stimulant in humans. The European Food Safety Authority (EFSA) recommended that caffeine intake should be below 400 mg/day for adults and 3 mg/kg bw/day for children and adolescents [14]. Tea is the major contributor to the daily caffeine intake of tea drinkers [12,33]. Typically, the transference of the caffeine in tea from leaves to infusion is confirmed to be up to 85.2% [12], and so there is about 60 mg of caffeine in a cup of tea. It is believed that a habitual daily intake of 500–600 mg caffeine (7 to 9 cup of tea) will result in significant health risks such as “caffeinism”, which refers to a syndrome characterized by a range of adverse reactions including diuresis, insomnia, headache, nausea, agitation, irritability, anxiety, arrhythmia, tachycardia, restlessness, tinnitus, muscle tremor, etc. [11]. The consumption of low-caffeine or caffeine-free teas would be an effective way to limit daily intake of caffeine. *C. ptilophylla* wild tea plants would be a good source of leaf materials for processing low caffeine or caffeine-free tea [24]. However, the present study shows that there was great differentiation in caffeine content between individuals in the seed-propagated population of *C. ptilophylla* plants, among which there were only a few non-caffeinated plants (Table 2). Tea plants are a self-incompatible crop [34]. The wild *C. ptilophylla* tea plants grew in natural fields with population disintegration, which could be divided into two demes: one containing theobromine plus caffeine and the other containing theobromine according the distribution pattern of purine alkaloids [35]. The seeds we collected on the *C. ptilophylla* wild tea plants might be from hybrid fruits produced by crossing the female gametes of *C. ptilophylla* with pollen from normal tea plants (*C. sinensis*). These factors suggest that it is impossible to obtain a pure, non-caffeinated offspring population from seeds collected on *C. ptilophylla* wild tea plants in an open filed. As *C. ptilophylla* is difficult to propagated by cutting, it would be a good choice to graft the scions of caffeine-free *C. ptilophylla* plants on the stocks of cultivated tea plant (*C. sinensis*) or *Camellia oleifera* [13]. If the plants are to be cultivated from seedlings, then the seed production plants must be isolated using nets during the flowering period (September to November) in order to prevent hybridization with the exotic pollen of common tea plants. Polyphenols are the prominent quality and health-beneficial compounds in tea, of which catechins are the most important. According to the published methods [28,29,31,32,36], eight components of catechins and four purine alkaloids were identified based on the retention time of reference compounds (Figure 2). For the normal cultivated tea plant (*C. sinensis*), EGCG is the most abundant component of catechins [31,32]. This study reveals that GCG is the most abundant component of catechins in the leaves of seedlings of *C. ptilophylla* wild plants (Table 3), which is consist with the published data [24,28,29,36,37]. EGCG is the most important bioactive in tea, but it is unstable and susceptible to oxidization under ambient conditions. GCG, an epimer counterpart of EGCG, however, is more chemically stable than EGCG [2]. The leaves of *C. ptilophylla* would be a good source of materials for preparing natural tea antioxidants, being more stable and more suitable for cosmetic ingredients than traditional tea antioxidants.

Concentrations of amino acids and total catechins are important tea quality indicators. Usually, leaves from cultivars containing a higher content of catechins and lower content of amino acids are suitable for processing black tea, while on the contrary, those with a higher level of amino acids and a medium level of catechins are suitable for processing green tea [31]. The leaves of *C. ptilophylla* wild plants are usually considered to be suitable for processing black tea because of their high contents of catechins [23,24,25]. Normally marketed green teas contained 3–6% of total amino acids and 90–150 mg/g of total catechins [21,22,32]. The present study shows that there were a few individual seed-propagated *C. ptilophylla* plants, such as KS2B, KS23A, and KS55B (Table 1 and Table 3), which contained higher levels of amino acids and medium levels of catechins, suggesting that they might be good candidates for further screening as green tea cultivars.

## 5. Conclusions

The chemical compositions of 39 individual seed-propagated plants from wild cocoa tea plant seeds were investigated. There was great variation in caffeine content among the tested plants, among which 18 plants (46% of total plants) grew leaves with caffeine contents less than 5 mg/g, the maximum limit of less-caffeinated tea. This suggests that propagation of cocoa tea seeds is not suitable for the production of less-caffeinated tea. Compared to normal tea cultivars (*C. sinensis*), leaves from cocoa tea plants are rich in GCG, a chemically more stable component of catechins than EGCG. Cocoa tea leaves would be a good source of GCG for use in the cosmetic industry. It was also found that cocoa tea leaves contained high levels of polyphenols and amino acids compared to green tea products, suggesting that they have the potential to be cultivated as high-quality black tea or green tea cultivars.

## Figures and Tables

**Table 1:** Contents of total polyphenols and amino acids in the dry tea leaves (% DW, mean ± SD, *n* = 3).

Plant No.	Total Polyphenols (EGCG Equivalent)	Amino Acids
KS1	31.96 ± 0.81 ^cdef①^	6.75 ± 0.23 ^ijk^
KS2	33.74 ± 1.13 ^b^	4.15 ± 0.03 ^u^
KS2B	27.87 ± 0.74 ^mn^	7.32 ± 0.12 ^gh^
KS3	31.36 ± 0.17 ^efghi^	6.22 ± 0.14 ^lmn^
KS4	28.99 ± 0.26 ^jklm^	6.51 ± 0.21 ^klm^
KS6	30.28 ± 0.52 ^ghijk^	6.97 ± 0.05 ^hij^
KS7	36.03 ± 0.95 ^a^	3.97 ± 0.11 ^u^
KS8	18.33 ± 0.54 ^q^	3.42 ± 0.05 ^v^
KS10	28.69 ± 0.57 ^klm^	5.66 ± 0.03 ^op^
KS17	27.00 ± 0.37 ^no^	8.25 ± 0.02 ^d^
KS21	27.90 ± 0.5 ^mn^	4.53 ± 0.16 ^t^
KS22	29.84 ± 0.36 ^ijkl^	6.60 ± 0.11 ^jkl^
KS23A	27.42 ± 0.36 ^mn^	10.40 ± 0.33 ^a^
KS27	30.06 ± 0.3 ^hijkl^	6.66 ± 0.32 ^ijk^
KS31	28.08 ± 0.55 ^mn^	5.91 ± 0.40 ^no^
KS32	31.54 ± 0.18 ^defgh^	7.98 ± 0.08 ^de^
KS33	32.50 ± 0.57 ^bcde^	6.25 ± 0.08 ^lmn^
KS34	35.44 ± 1.69 ^a^	5.42 ± 0.10 ^pq^
KS35	27.72 ± 1.11 ^mn^	3.25 ± 0.03 ^v^
KS36	18.32 ± 0.34 ^q^	3.41 ± 0.03 ^v^
KS37	25.86 ± 0.61 ^op^	4.89 ± 0.12 ^rst^
KS38	33.25 ± 0.82 ^bc^	4.59 ± 0.08 ^st^
KS40	32.05 ± 0.9 ^cdef^	5.10 ± 0.15 ^qr^
KS41	28.74 ± 0.74 ^klm^	8.91 ± 0.26 ^c^
KS43	25.50 ± 0.24 ^p^	6.02 ± 0.08 ^no^
KS44	31.75 ± 0.59 ^cdefg^	6.90 ± 0.25 ^ijk^
KS45	32.00 ± 0.55 ^cdef^	5.44 ± 0.15 ^pq^
KS47	29.84 ± 1.02 ^ijkl^	5.49 ± 0.08 ^pq^
KS48	28.54 ± 0.55 ^lmn^	4.96 ± 0.11 ^rs^
KS49	25.27 ± 0.35 ^p^	5.98 ± 0.19 ^no^
KS50	24.35 ± 0.08 ^p^	6.71 ± 0.13 ^ijk^
KS51	33.07 ± 1.16 ^bcd^	6.14 ± 0.13 ^mn^
KS53	28.58 ± 1.05 ^lmn^	7.06 ± 0.41 ^hi^
KS54	32.90 ± 0.16 ^bcde^	6.56 ± 0.18 ^jkl^
KS55A	30.40 ± 0.39 ^fghij^	7.45 ± 0.14 ^fg^
KS55B	25.16 ± 0.83 ^p^	8.08 ± 0.21 ^de^
KS59	33.78 ± 1.06 ^b^	6.06 ± 0.06 ^no^
KS62	30.70 ± 0.94 ^fghi^	7.76 ± 0.16 ^ef^
KS65	32.67 ± 0.3 ^bcde^	9.56 ± 0.42 ^b^
Mean	29.42 ± 3.88	6.24 ± 1.63
CV (%) ^②^	13.20	26.10

^①^: Data with different lowercase superscript letters in the same column were significantly different at *p* < 0.05. ^②^: CV, coefficient of variation.

**Table 2:** Contents of alkaloids in the dry tea leaves (mg/g DW, Mean ± SD, *n* = 3).

Plant No.	Caffeine	Theacrine	Theobromine	Theophylline	Total
KS1	52.69 ± 3.54 ^a①^	6.09 ± 0.44 ^e^	11.66 ± 0.73 ^op^	0	70.45 ± 4.69 ^a^
KS2	1.24 ± 0.05 ^m^	4.17 ± 0.04 ^j^	52.49 ± 1.26 ^c^	0	57.89 ± 1.36 ^cd^
KS2B	28.33 ± 0.28 ^ij^	3.79 ± 0.03 ^k^	9.71 ± 0.16 ^q^	0	41.83 ± 0.46 ^op^
KS3	39.44 ± 0.31 ^fg^	1.71 ± 0.02 ^pqr^	14.69 ± 0.17 ^mn^	0	55.84 ± 0.46 ^de^
KS4	34.06 ± 0.23 ^h^	5.77 ± 0.03 ^fg^	16.18 ± 0.14 ^m^	0	56.02 ± 0.38 ^de^
KS6	1.39 ± 0.03 ^m^	1.09 ± 0.01 ^t^	55.45 ± 1.12 ^b^	0	57.93 ± 1.11 ^cd^
KS7	0.98 ± 0.03 ^m^	2.34 ± 0.04 ^o^	38.69 ± 0.52 ^ij^	0	42.01 ± 0.58 ^op^
KS8	21.37 ± 0.97 ^l^	5.08 ± 0.20 ^h^	0.93 ± 0.01 ^u^	0.11 ± 0.05 ^a^	27.50 ± 1.22 ^r^
KS10	45.74 ± 2.53 ^b^	2.96 ± 0.02 ^n^	6.94 ± 0.12 ^r^	0.08 ± 0.12 ^ab^	55.73 ± 2.50 ^de^
KS17	35.60 ± 0.87 ^h^	2.59 ± 0.05 ^o^	9.99 ± 0.24 ^pq^	0	48.17 ± 1.15 ^jklm^
KS21	0.96 ± 0.01 ^m^	1.81 ± 0.09 ^pq^	37.03 ± 0.21 ^jk^	0	39.80 ± 0.28 ^p^
KS22	38.34 ± 1.42 ^g^	3.29 ± 0.16 ^lm^	18.71 ± 0.25 ^l^	0	60.34 ± 1.61 ^c^
KS23A	0.60 ± 0.08 ^m^	4.83 ± 0.17 ^hi^	39.13 ± 1.16 ^i^	0	44.55 ± 1.25 ^no^
KS27	0	1.61 ± 0.02 ^pqr^	47.55 ± 0.50 ^ef^	0	49.17 ± 0.48 ^ijkl^
KS31	33.46 ± 0.09 ^h^	1.73 ± 0.03 ^pqr^	13.08 ± 0.10 ^no^	0	48.28 ± 0.12 ^jklm^
KS32	45.09 ± 0.28 ^bc^	5.78 ± 0.01 ^f^	3.72 ± 0.01 ^s^	0	54.59 ± 0.28 ^defg^
KS33	0.80 ± 0.01 ^m^	3.55 ± 0.03 ^kl^	47.03 ± 0.43 ^fg^	0	51.38 ± 0.47 ^ghij^
KS34	42.69 ± 1.52 ^de^	5.46 ± 0.24 ^g^	15.29 ± 0.56 ^m^	0	63.45 ± 2.32 ^b^
KS35	24.76 ± 2.66 ^k^	3.65 ± 0.18 ^k^	1.02 ± 0.05 ^u^	0.15 ± 0.01 ^a^	29.57 ± 2.70 ^r^
KS36	0	1.12 ± 0.07 ^st^	38.06 ± 2.95 ^ij^	0	39.18 ± 3.02 ^p^
KS37	0	0.94 ± 0.08 ^t^	43.34 ± 0.42 ^h^	0.12 ± 0.09 ^a^	44.40 ± 0.26 ^no^
KS38	0.27 ± 0.38 ^m^	1.92 ± 0.06 ^p^	45.45 ± 1.60 ^g^	0	47.64 ± 1.99 ^klmn^
KS40	1.90 ± 0.04 ^m^	1.48 ± 0.01 ^qr^	43.54 ± 0.74 ^h^	0	46.92 ± 0.78 ^lmn^
KS41	40.73 ± 1.90 ^ef^	1.84 ± 0.08 ^pq^	3.39 ± 0.17 ^st^	0	45.96 ± 2.16 ^lmn^
KS43	43.37 ± 1.37 ^cd^	4.68 ± 0.12 ^i^	6.07 ± 0.17 ^r^	0	54.13 ± 1.66 ^efgh^
KS44	38.06 ± 0.70 ^g^	4.12 ± 0.05 ^j^	2.49 ± 0.03 ^stu^	0	44.68 ± 0.77 ^no^
KS45	0.62 ± 0.01 ^m^	7.77 ± 0.12 ^b^	36.2 ± 0.58 ^k^	0	44.59 ± 0.71 ^no^
KS47	1.11 ± 0.03 ^m^	1.50 ± 0.05 ^qr^	50.48 ± 1.24 ^d^	0.11 ± 0.15 ^a^	53.19 ± 1.12 ^efgh^
KS48	1.51 ± 0.04 ^m^	1.49 ± 0.03 ^qr^	42.09 ± 1.02 ^h^	0	45.09 ± 1.08 ^mno^
KS49	30.18 ± 0.40 ^i^	1.03 ± 0.23 ^t^	2.09 ± 0.12 ^stu^	0.09 ± 0.12 ^ab^	33.38 ± 0.56 ^q^
KS50	27.69 ± 0.23 ^j^	0.91 ± 0.14 ^t^	1.69 ± 0.04 ^tu^	0	30.28 ± 0.15 ^r^
KS51	38.81 ± 0.63 ^fg^	7.07 ± 0.10 ^c^	5.94 ± 0.30 ^r^	0	51.82 ± 0.92 ^fghi^
KS53	42.99 ± 1.45 ^cde^	6.57 ± 0.23 ^d^	1.32 ± 0.05 ^u^	0	50.89 ± 1.73 ^hijk^
KS54	0	3.20 ± 0.06 ^mn^	49.06 ± 0.72 ^de^	0	52.26 ± 0.78 ^fghi^
KS55A	42.49 ± 0.89 ^de^	1.00 ± 0.01 ^t^	11.35 ± 0.36 ^opq^	0.06 ± 0.05 ^ab^	54.91 ± 1.20 ^def^
KS55B	24.03 ± 0.88 ^k^	1.42 ± 0.01 ^rs^	1.58 ± 0.04 ^tu^	0	27.03 ± 0.90 ^r^
KS59	0.10 ± 0.02 ^m^	15.92 ± 0.57 ^a^	53.57 ± 1.04 ^c^	0	69.60 ± 1.58 ^a^
KS62	0	1.70 ± 0.04 ^pqr^	42.72 ± 1.32 ^h^	0	44.42 ± 1.35 ^no^
KS65	1.23 ± 0.03 ^m^	1.49 ± 0.03 ^qr^	65.39 ± 1.54 ^a^	0	68.10 ± 1.60 ^a^
Mean	20.07 ± 19.11	3.45 ± 2.83	25.26 ± 20.50	0.02 ± 0.04	48.79 ± 10.70
CV (%) ^②^	95.25	81.96	81.16	225.58	21.94

^①^: Data with different lowercase superscript letters in the same column were significantly different at *p* < 0.05. ^②^: CV, coefficient of variation.

**Table 3:** Contents of catechins in the dry tea leaves (mg/g DW, Mean ± SD, *n* = 3).

Plant No.	GCG	EGCG	ECG	C	CG	GC	EGC	EC	Total Catechins
KS1	110.16 ± 8.85 ^fgh①^	54.81 ± 4.04 ^d^	26.76 ± 1.98 ^e^	13.18 ± 0.73 ^st^	1.06 ± 0.17 ^rst^	18.78 ± 1.14 ^h^	10.87 ± 0.91 ^de^	4.15 ± 0.26 ^defgh^	239.78 ± 17.96 ^c^
KS2	108.37 ± 3.50 ^ghi^	32.23 ± 0.79 ^i^	17.12 ± 0.63 ^l^	17.65 ± 0.16 ^pq^	3.75 ± 0.13 ^no^	2.94 ± 0.03 ^p^	1.67 ± 0.04 ^hi^	3.16 ± 0.18 ^fghij^	186.88 ± 5.04 ^jklmn^
KS2B	44.24 ± 0.65 ^op^	25.41 ± 0.24 ^k^	15.42 ± 0.17 ^m^	14.24 ± 0.13 ^rst^	16.38 ± 0.17 ^e^	18.88 ± 0.06 ^h^	0	1.79 ± 0.16 ^ijkl^	136.38 ± 1.16 ^rs^
KS3	135.73 ± 1.89 ^cd^	4.38 ± 0.06 ^nop^	21.94 ± 0.38 ^g^	21.01 ± 0.05 ^klm^	23.46 ± 0.20 ^b^	32.51 ± 0.21 ^c^	0	0.97 ± 0.04 ^jkl^	239.99 ± 2.56 ^c^
KS4	81.02 ± 0.52 ^l^	36.14 ± 0.29 ^gh^	10.92 ± 0.15 ^n^	11.28 ± 0.06 ^u^	1.99 ± 0.08 ^pq^	16.31 ± 0.02 ^i^	8.79 ± 0.15 ^f^	3.65 ± 0.07 ^efghi^	170.10 ± 1.11 ^op^
KS6	136.36 ± 2.76 ^cd^	3.69 ± 0.06 ^nop^	33.32 ± 0.88 ^b^	24.57 ± 0.16 ^gh^	25.78 ± 0.66 ^a^	1.88 ± 0.02 ^p^	0	0	225.60 ± 4.52 ^def^
KS7	141.86 ± 3.38 ^c^	6.09 ± 0.10 ^n^	40.32 ± 1.06 ^a^	28.92 ± 0.44 ^d^	17.41 ± 0.29 ^d^	20.27 ± 0.38 ^g^	0	2.25 ± 0.06 ^ghijk^	257.11 ± 5.52 ^b^
KS8	17.71 ± 0.96 ^r^	16.95 ± 1.02 ^l^	6.88 ± 0.24 ^o^	2.57 ± 0.10 ^x^	0.82 ± 0.05 ^st^	8.53 ± 0.23 ^m^	11.00 ± 0.71 ^d^	6.12 ± 0.30 ^d^	70.58 ± 2.24 ^u^
KS10	103.93 ± 1.77 ^hij^	16.09 ± 0.27 ^l^	26.32 ± 0.65 ^e^	16.85 ± 0.15 ^q^	10.37 ± 0.15 ^fg^	16.23 ± 0.16 ^i^	2.79 ± 2.80 ^h^	4.39 ± 5.45 ^defg^	190.45 ± 13.68 ^jkl^
KS17	97.48 ± 2.33 ^jk^	17.95 ± 0.21 ^l^	10.93 ± 0.22 ^n^	19.48 ± 0.61 ^mno^	3.12 ± 0.09 ^o^	19.76 ± 0.37 ^gh^	6.00 ± 0.11 ^g^	0.80 ± 0.10 ^kl^	175.54 ± 3.78 ^nop^
KS21	84.08 ± 0.65 ^l^	3.75 ± 0.16 ^nop^	15.15 ± 0.24 ^m^	23.58 ± 0.48 ^hi^	10.72 ± 0.19 ^f^	10.96 ± 0.26 ^l^	0	2.00 ± 0.29 ^hijkl^	150.25 ± 0.53 ^q^
KS22	92.33 ± 2.59 ^k^	28.56 ± 1.04 ^j^	15.05 ± 0.95 ^m^	19.16 ± 0.42 ^no^	10.05 ± 0.20 ^gh^	11.55 ± 0.93 ^l^	10.31 ± 1.07 ^de^	1.66 ± 0.06 ^ijkl^	188.66 ± 6.49 ^jklmn^
KS23A	46.66 ± 1.68 ^o^	25.85 ± 1.00 ^k^	17.34 ± 0.75 ^kl^	16.95 ± 0.46 ^q^	7.87 ± 0.34 ^j^	13.20 ± 0.35 ^k^	0	3.29 ± 0.14 ^fghi^	131.16 ± 4.46 ^rs^
KS27	130.99 ± 2.16 ^d^	3.79 ± 0.09 ^nop^	20.21 ± 0.34 ^hi^	35.21 ± 0.28 ^c^	19.09 ± 0.35 ^c^	30.30 ± 0.33 ^d^	0	14.27 ± 0.10 ^bc^	253.86 ± 2.84 ^b^
KS31	134.19 ± 0.99 ^d^	3.40 ± 0.27 ^op^	15.21 ± 0.12 ^m^	17.30 ± 0.15 ^q^	9.48 ± 0.11 ^hi^	20.03 ± 0.07 ^gh^	0	0	199.61 ± 0.72 ^hij^
KS32	78.23 ± 0.33 ^l^	31.28 ± 0.11 ^i^	14.20 ± 0.09 ^m^	15.12 ± 0.11 ^r^	1.16 ± 0.01 ^rst^	20.33 ± 0.11 ^g^	9.62 ± 0.02 ^ef^	2.29 ± 0.02 ^ghijk^	174.38 ± 3.34 ^nop^
KS33	112.42 ± 0.94 ^fg^	32.46 ± 0.35 ^i^	23.83 ± 0.26 ^f^	14.66 ± 0.14 ^rs^	5.74 ± 0.06 ^l^	2.19 ± 0.09 ^p^	1.21 ± 0.04 ^ij^	2.10 ± 0.16 ^hijkl^	194.62 ± 1.49 ^ijk^
KS34	108.80 ± 5.69 ^ghi^	34.97 ± 1.69 ^h^	20.69 ± 1.10 ^gh^	19.05 ± 0.73 ^op^	3.73 ± 0.21 ^no^	22.93 ± 0.87 ^f^	10.06 ± 0.55 ^de^	2.41 ± 0.23 ^ghijk^	225.61 ± 13.08 ^def^
KS35	28.00 ± 1.16 ^q^	11.16 ± 0.52 ^m^	15.04 ± 0.66 ^m^	7.03 ± 0.31 ^w^	1.45 ± 0.19 ^qrs^	6.54 ± 0.36 ^n^	0	5.55 ± 0.07 ^de^	76.38 ± 4.20 ^u^
KS36	68.96 ± 5.02 ^m^	2.75 ± 0.20 ^op^	6.27 ± 0.47 ^o^	19.85 ± 1.23 ^mno^	5.41 ± 0.38 ^l^	10.93 ± 0.69 ^l^	0	1.87 ± 0.17 ^ijkl^	116.05 ± 8.07 ^t^
KS37	123.17 ± 0.39 ^e^	9.14 ± 0.02 ^m^	14.40 ± 0.09 ^m^	21.44 ± 0.07 ^jkl^	4.59 ± 0.10 ^m^	48.64 ± 1.11 ^b^	6.18 ± 0.52 ^g^	2.54 ± 0.16 ^fghijk^	230.11 ± 1.55 ^cde^
KS38	153.94 ± 4.88 ^b^	4.97 ± 0.20 ^no^	41.46 ± 1.19 ^a^	36.53 ± 2.10 ^c^	25.33 ± 0.59 ^a^	33.56 ± 1.05 ^c^	0	21.00 ± 0.74 ^a^	316.79 ± 7.15 ^a^
KS40	113.30 ± 2.27 ^fg^	3.85 ± 0.02 ^nop^	31.66 ± 0.69 ^c^	22.33 ± 0.21 ^ijk^	16.98 ± 0.36 ^de^	1.88 ± 0.05 ^p^	0	0	190.01 ± 3.49 ^jklm^
KS41	116.86 ± 6.07 ^f^	4.89 ± 0.23 ^no^	16.90 ± 0.97 ^l^	26.43 ± 1.25 ^ef^	16.46 ± 0.89 ^e^	21.97 ± 0.89 ^f^	0	15.44 ± 0.83 ^b^	218.95 ± 11.11 ^efg^
KS43	79.94 ± 2.58 ^l^	38.32 ± 1.24 ^g^	18.96 ± 0.57 ^ij^	10.23 ± 0.21 ^uv^	2.19 ± 0.11 ^p^	13.48 ± 0.36 ^k^	12.74 ± 0.22 ^c^	1.74 ± 0.12 ^ijkl^	175.74 ± 7.40 ^mnop^
KS44	82.33 ± 1.98 ^l^	26.60 ± 0.52 ^jk^	14.90 ± 0.31 ^m^	21.75 ± 0.36 ^jkl^	1.40 ± 0.04 ^qrs^	14.64 ± 0.26 ^j^	6.08 ± 0.12 ^g^	4.68 ± 0.13 ^def^	172.38 ± 3.64 ^op^
KS45	39.26 ± 0.63 ^p^	47.32 ± 0.86 ^e^	20.43 ± 0.34 ^h^	8.95 ± 0.10 ^v^	1.64 ± 0.02 ^pqr^	13.06 ± 0.16 ^k^	7.31 ± 0.29 ^g^	5.68 ± 0.07 ^de^	143.65 ± 2.32 ^qr^
KS47	114.27 ± 3.37 ^fg^	2.65 ± 0.08 ^op^	18.88 ± 0.77 ^ijk^	28.87 ± 0.29 ^d^	19.59 ± 0.57 ^c^	25.43 ± 0.60 ^e^	0	0	209.70 ± 5.54 ^gh^
KS48	113.22 ± 3.43 ^fg^	3.15 ± 0.10 ^op^	12.35 ± 0.5 ^n^	22.95 ± 0.19 ^ij^	7.17 ± 0.28 ^k^	18.97 ± 0.15 ^h^	0	0	177.81 ± 4.43 ^lmnop^
KS49	2.12 ± 0.06 ^s^	79.15 ± 1.62 ^b^	18.83 ± 0.46 ^ijk^	0.97 ± 0.14 ^y^	0.14 ± 0.01 ^u^	3.06 ± 1.05 ^p^	49.99 ± 1.37 ^a^	12.59 ± 0.34 ^c^	166.86 ± 4.53 ^op^
KS50	2.02 ± 0.03 ^s^	74.3 ± 1.67 ^c^	17.72 ± 0.28 ^jkl^	1.13 ± 0.21 ^y^	0.13 ± 0.01 ^u^	5.16 ± 0.56 ^o^	50.41 ± 1.09 ^a^	12.97 ± 0.49 ^c^	163.84 ± 4.19 ^p^
KS51	95.39 ± 2.67 ^k^	48.23 ± 1.10 ^e^	18.40 ± 0.39 ^jkl^	13.91 ± 0.13 ^rst^	0.91 ± 0.04 ^st^	20.40 ± 0.30 ^g^	8.56 ± 0.02 ^f^	6.06 ± 0.14 ^d^	213.47 ± 6.55 ^fg^
KS53	66.19 ± 2.13 ^m^	44.97 ± 1.54 ^f^	24.43 ± 0.85 ^f^	12.99 ± 0.48 ^t^	0.94 ± 0.04 ^st^	10.53 ± 0.31 ^l^	7.05 ± 0.2 ^g^	3.21 ± 0.20 ^fghi^	170.32 ± 5.60 ^op^
KS54	103.19 ± 1.64 ^ij^	25.47 ± 0.44 ^k^	11.98 ± 0.22 ^n^	27.38 ± 0.47 ^e^	5.58 ± 0.13 ^l^	2.23 ± 0.12 ^p^	0	2.96 ± 0.22 ^fghijk^	178.81 ± 2.96 l^mno^
KS55A	14.29 ± 0.38 ^r^	122.63 ± 3.74 ^a^	29.84 ± 0.99 ^d^	2.79 ± 0.07 ^x^	0.65 ± 0.03 ^tu^	6.30 ± 0.15 ^no^	28.53 ± 0.43 ^b^	2.59 ± 0.28 ^fghijk^	207.62 ± 5.00 ^ghi^
KS55B	55.51 ± 2.38 ^n^	2.09 ± 0.03 ^p^	11.23 ± 0.61 ^n^	20.63 ± 0.42 ^lmn^	4.32 ± 0.25 ^mn^	30.22 ± 1.10 ^d^	0	1.55 ± 0.21 ^ijkl^	125.56 ± 4.61 ^st^
KS59	54.47 ± 1.21 ^n^	2.70 ± 0.20 ^op^	2.10 ± 0.75 ^p^	44.23 ± 2.70 ^a^	19.47 ± 0.55 ^c^	57.02 ± 1.32 ^a^	0	0.85 ± 0.50 ^kl^	180.85 ± 4.36 ^klmno^
KS62	101.88 ± 3.04 ^j^	3.49 ± 0.11 ^nop^	18.75 ± 0.65 ^ijk^	40.55 ± 0.96 ^b^	8.92 ± 0.41 ^i^	21.78 ± 0.60 ^f^	0	13.33 ± 0.35 ^c^	208.69 ± 6.12 ^gh^
KS65	161.45 ± 4.30 ^a^	4.56 ± 0.09 ^nop^	33.53 ± 1.04 ^b^	25.82 ± 0.51 ^fg^	7.57 ± 0.22 ^jk^	0	0	0	232.93 ± 6.15 ^cd^
Mean	88.57 ± 41.69	24.11 ± 25.97	19.22 ± 8.67	19.17 ± 10.08	8.28 ± 7.78	16.75 ± 12.45	6.39 ±11.86	4.36 ± 5.00	186.85 ± 48.12
CV(%)^②^	47.07	107.71	45.12	52.59	93.94	74.29	185.55	114.82	25.75

^①^: Data with different lowercase superscript letters in the same column were significantly different at *p* < 0.05. ^②^: CV, coefficient of variation.

## Data Availability

Data are available on request.
